# Correlation between albumin levels during the third trimester and the risk of postpartum levator ani muscle rupture

**DOI:** 10.1515/med-2025-1209

**Published:** 2025-06-09

**Authors:** Yueyun Cai, Yuting Cai, Yuanling Hu, Shengkai Lu, Yueqing Lin

**Affiliations:** Department of Obstetrical, Zhangzhou Affiliated Hospital of Fujian medical University, Fujian, 363000, P.R. China; Department of Obstetrical, Zhangzhou Affiliated Hospital of Fujian medical University, 193 Shuixian Street, Longwen District, Zhangzhou, Fujian, 363000, P.R. China

**Keywords:** week before delivery, albumin levels, levator ani muscle rupture, postpartum, risk factors

## Abstract

**Objective:**

To investigate the correlation between albumin levels during the third trimester and the risk of postpartum levator ani muscle rupture.

**Methods:**

A retrospective analysis of 410 parturients undergoing vaginal delivery at Zhangzhou Hospital Affiliated to Fujian Medical University was conducted. Parturients were classified into low-albumin (<35 g/L) and normal-albumin (≥35 g/L) groups based on their albumin levels during the third trimester. Data on levator ani muscle rupture and various clinical parameters were collected and analyzed using multivariate binary logistic regression.

**Results:**

The low-albumin group comprised 38.29% of participants and had a significantly higher incidence of levator ani muscle rupture (53.50%) compared to the normal-albumin group (33.20%; *P* < 0.05). Albumin levels strongly correlated with rupture risk (*r* = 0.193, *P* < 0.001). Multivariate analysis showed low albumin levels were an independent risk factor (OR = 2.286, 95% CI: 1.259–4.152). Other independent risk factors included forceps-assisted delivery, BMI ≥ 24 kg/m², age ≥ 35 years, and second stage of labor duration ≥ 120 min (all *P* < 0.05).

**Conclusion:**

Decreased albumin levels are an independent risk factor for postpartum levator ani muscle rupture, suggesting a potential role in increasing the risk of muscle injury.

## 1 Introduction

The levator ani muscle is an essential component of the pelvic floor muscles, playing a crucial role in supporting pelvic organs and maintaining their normal functions. In clinical practice, levator ani muscle injury is often considered one of the primary factors contributing to postpartum pelvic floor dysfunction [1,2]. The parturients were categorized into a low-albumin group (<35 g/L) and a normal-albumin group (≥35 g/L) based on their albumin levels. Furthermore, these problems increase the economic burden on families and society, significantly elevating medical expenses and nursing costs [3]. Current research on levator ani muscle rupture primarily focuses on the risk factors for its occurrence. Studies have shown that age, mode of delivery, and the physical condition of the parturient may be closely related to the occurrence of levator ani muscle rupture [4,5]. However, the specific relationship between maternal physiological indicators and levator ani muscle rupture remains unclear, particularly regarding the factor of albumin level, which has been relatively limited in research. Albumin, the most abundant protein in the human plasma synthesized by the liver, is mainly classified into serum albumin, urinary albumin, cerebrospinal fluid albumin, etc. Among them, serum albumin is commonly used for clinical assessment, with normal levels maintained at 40–55 g/L. For pregnant women, influenced by blood volume changes during pregnancy, albumin concentration is relatively lower than that of non-pregnant women, often hovering near the lower limit of the normal range, mostly between 30 and 55 g/L. Additionally, pregnancy-related complications such as hypertension and kidney disease may result in excessive albumin loss in pregnant women, leading to decreased albumin levels [6].

Albumin, the most abundant protein in plasma, plays a pivotal role in tissue repair and inflammation regulation. It promotes cell proliferation, migration, and differentiation, essential for wound healing [7]. Additionally, albumin acts as an antioxidant and anti-inflammatory factor, protecting cells and tissues from oxidative stress and inflammatory damage. Besides maintaining colloid osmotic pressure in the body, albumin also plays a key role in tissue repair, inflammatory response, and immune regulation. Existing studies have shown that hypoalbuminemia is associated with poor prognosis in various diseases (such as liver cirrhosis and renal diseases) and is considered a potential risk factor in multiple obstetric complications; however, its role in levator ani muscle rupture requires further exploration [8].

Based on this, lower albumin levels may lead to excessive local inflammatory responses and delayed repair, thereby increasing the risk of tissue rupture, providing a scientific basis for nutritional intervention, rehabilitation treatment, and functional recovery in postpartum women. We hope that this study will offer new insights into the clinical prevention and treatment of postpartum pelvic floor dysfunction, thereby improving the health status and quality of life of postpartum women.

## 2 Materials and methods

### 2.1 Data source

This study collected 410 parturients who underwent vaginal delivery in the Obstetrics Department of Zhangzhou Affiliated Hospital of Fujian medical University from January 2020 to June 2023. The study was approved by the hospital’s ethics committee (Ethics Number: 2024LWB351). All participants underwent ultrasonic pelvic floor function examinations. Data collection and collation were jointly managed by two researchers.

### 2.2 Inclusion and exclusion criteria

The inclusion and exclusion criteria for this study are summarized in Table 1.

**Table 1:** Inclusion and exclusion criteria

Category	Criteria
Inclusion criteria	1. Primiparous women who had received standardized prenatal care and delivered at our hospital
	2. Singleton term vaginal delivery
	3. Postpartum period of 6–8 weeks after the lochia had cleared
	4. Ability to cooperate in completing pelvic floor muscle contraction (pelvic organs move towards the head and abdomen during contraction, with a reduction in the area of the levator ani hiatus, and contraction duration ≥3 s) and Valsalva maneuver (deep inhalation followed by breath-holding and exerting maximum force downwards to achieve maximum abdominal pressure, during which pelvic organs move toward the dorsal and caudal sides, the area of the levator ani hiatus increases, and the duration is ≥6 s)
Exclusion criteria	1. History of pelvic, vaginal surgery, or trauma
	2. History of prenatal urinary incontinence
	3. History of diseases that increase abdominal pressure, such as chronic cough or chronic constipation
	4. Chronic underlying diseases such as cardiovascular and cerebrovascular diseases, endocrine disorders, liver or kidney diseases
	5. Patients with mental illnesses that impair communication
	6. Patients with malignant tumors

Inclusion criteria included: (1) primiparous women who had received standardized prenatal care and delivered at our hospital, (2) singleton term vaginal delivery, (3) postpartum period of 6–8 weeks after the lochia had cleared, (4) ability to cooperate in completing pelvic floor muscle contraction (where pelvic organs move toward the head and abdomen during contraction, with a reduction in the area of the levator ani hiatus, and contraction duration ≥3 s) and the Valsalva maneuver (the most effective action, involving deep inhalation followed by breath-holding and exerting maximum force downward to achieve maximum abdominal pressure, during which pelvic organs move toward the dorsal and caudal sides, the area of the levator ani hiatus increases, and the duration is ≥6 s).

Exclusion criteria included: (1) history of pelvic, vaginal surgery, or trauma; (2) history of prenatal urinary incontinence; (3) history of diseases that increase abdominal pressure, such as chronic cough or chronic constipation; (4) chronic underlying diseases such as cardiovascular and cerebrovascular diseases, endocrine disorders, liver or kidney diseases; (5) patients with mental illnesses that impair communication; and (6) patients with malignant tumors.

### 2.3 Examination methods

Diagnosis of levator ani rupture: Color Doppler ultrasonic diagnostic equipment (Mindray resona8; probes, L14-5WU, SC5-1U, D8-2U; software packages: pelvic floor application software, ipaae+ advanced volumetric tomography, SCV+ advanced volumetric thick-slice imaging, intelligent pelvic floor ultrasound solution) was used for routine three-dimensional ultrasound examination of the perineal pelvic floor. Prior to the examination, patients were instructed to empty their bladder and intestines. During the examination, patients were positioned in the lithotomy position, with coupling agent applied to a disposable probe cover, which was then placed over the three-dimensional probe and positioned on the perineum. The pelvic floor structure was observed through different cross-sectional views. The TUI mode was selected to acquire and collect image information from the foot to the head in the reference plane. Three consecutive images were captured to observe the presence of complete or partial discontinuity of the levator ani muscle fibers as a diagnostic criterion.

Albumin detection method: Although Chinese guidelines do not mandatorily require albumin testing, it is routinely included in liver function tests performed at our hospital. Therefore, a 3 mL venous blood sample was collected in the morning after an overnight fast during the third trimester. The sample was centrifuged at 2,800 r/min for 12 min, and the supernatant was used to measure albumin levels using a Beckman AU5400 fully automatic biochemical analyzer (USA).

### 2.4 Observation indicators

The following data were collected: maternal age at delivery; body mass index (BMI), fundal height, abdominal circumference, and biparietal diameter in the week before delivery; gestational age at delivery; duration of the first, second, and third stages of labor; whether episiotomy was performed; presence of perineal tears; neonatal weight; use of forceps during delivery; and use of analgesia during delivery. Blood test results for hemoglobin, albumin, calcium, and magnesium were also collected from the week before delivery.

### 2.5 Statistical methods

Statistical analysis was performed using SPSS 26.0 software. First, normality and homogeneity of variance tests were conducted on the measurement data. For measurement data that followed a normal distribution and had homogeneous variances, mean ± standard deviation (±s) was used for description, and independent sample *t*-tests were used for comparisons between groups. For data with heterogeneous variances or non-normal distributions, the median (interquartile range) M (P25, P75) was used for description, and non-parametric tests such as the Mann–Whitney *U*-test were applied. Count data were expressed as frequencies and proportions (%), and comparisons between groups were made using the chi-square test. For situations with small sample sizes or low expected frequencies, the Fisher’s exact test was used. Difference tests were conducted, along with univariate analysis and multivariate logistic regression analysis, to identify independent risk factors.


**Ethical approval:** The study was approved by Zhangzhou Affiliated Hospital of Fujian medical University (2024LWB351).

## 3 Results

### 3.1 Incidence of levator ani muscle avulsion (LAMA) and its correlation with albumin levels

Among the 410 participants, the group with low albumin levels accounted for 38.29% (157/410) of the total, while the group with normal albumin levels accounted for 61.71% (253/410). The research findings indicated that the incidence of LAMA in the low albumin group was 53.50% (84/157), significantly higher than the 33.20% (84/253) observed in the normal albumin group, with a statistically significant difference (*P* < 0.001). Spearman correlation analysis revealed a strong correlation between albumin levels and the risk of LAMA (*r* = 0.193, *P* < 0.001), as shown in Table 2.

**Table 2:** Comparison of parturient data between the two groups

	Low albumin group (*n* = 157)	Normal albumin group (*n* = 253)	\[t\text{/}Z\text{/}{\chi }^{2}]\]	*P*
Age (years)	28.84 ± 0.36	27.08 ± 0.20	4.522	<0.001
BMI at delivery (kg/m²)	25.30 (23.63,27.62)	21.69 (19.26,24.66)	8.068	<0.001
Fundal height (cm)	34 (33,34)	34 (33,34)	0.308	0.758
Abdominal circumference (cm)	96 (92,100)	98 (95,102)	1.480	0.139
Biparietal diameter of fetus (cm)	9.50 (9.20,9.80)	9.50 (9.20,9.90)	0.109	0.913
Duration of first stage of labor (min)	590 (371.25,800)	405 (238.75,675)	4.105	<0.001
Duration of second stage of labor (min)	64.50 (34,116)	36 (17,59)	6.051	<0.001
Duration of third stage of labor (min)	9 (7,10)	9 (7,10)	0.056	0.956
Episiotomy (*n*, %)	77 (49.04)	94 (37.15)	5.634	0.018
Perineal laceration (*n*, %)	65 (41.40)	156 (61.66)	16.002	<0.001
Forceps delivery (*n*, %)	68 (43.31)	18 (7.11)	76.580	<0.001
Labor analgesia (*n*, %)	61 (38.85)	87 (34.39)	0.838	0.360
Neonatal weight (g)	3224.55 ± 35.10	3199.16 ± 20.59	0.637	0.525
Hemoglobin (g/L)	124 (118,131)	125 (118,133)	0.747	0.455
Albumin (g/L)	34.75 (32.70,36.38)	36.25 (34.20,38.13)	4.829	<0.001
Calcium (mmol/L)	2.23 (2.17,2.29)	2.25 (2.18,2.34)	2.223	0.026
Age (years)	0.818 (0.760,0.853)	0.800 (0.756,0.838)	1.730	0.084

### 3.2 Clinical and laboratory characteristics of the LAMA group and the control group

There were no statistically significant differences in uterine height, abdominal circumference, biparietal diameter of the fetus, third stage of labor, labor analgesia, hemoglobin levels, or magnesium ion levels between the two groups of parturients (*P* > 0.05). However, statistically significant differences were observed in age, BMI at delivery, rate of episiotomy, rate of perineal tear, rate of forceps delivery, duration of the first stage of labor, duration of the second stage of labor, albumin levels, and calcium ion levels between the two groups (*P* < 0.05). In the low albumin group, parturients were older, had higher BMI at delivery, longer durations of the first and second stages of labor, higher rates of episiotomy, and higher rates of forceps delivery compared to the normal albumin group (*P* < 0.05). The LAMA group had higher rates of perineal tear and lower levels of albumin and calcium ions compared to the control group (*P* < 0.05), as presented in Table 1.

### 3.3 Univariate analysis and multivariate logistic regression analysis

Univariate logistic regression analysis revealed that low albumin levels (<35 g/L) were significantly associated with an increased risk of LAMA. Specifically, the risk of LAMA in the low albumin group was 2.391 times higher than that in the normal albumin group (95% CI: 1.535–3.725, *P* < 0.001). This finding suggests that low albumin levels may be an independent risk factor for LAMA, and decreased albumin levels may increase the risk of this condition. Additionally, factors such as age ≥35 years, BMI ≥ 24 kg/m^2^ at delivery, first stage of labor duration ≥480 min, second stage of labor duration ≥120 min, episiotomy, perineal tear, and the use of forceps were also significantly associated with the risk of LAMA, as shown in Table 3.

**Table 3:** Univariate analysis and multivariate logistic regression analysis

Variable	Grouping	Univariate analysis			Multivariate logistic regression analysis		
		*β*	OR (95%CI)	*P*	*β*	OR (95%CI)	*P*
Albumin	≥35 g/L*						
	<35 g/L	0.872	2.391 (1.535–3.725)	<0.001	0.827	2.286 (1.259–4.152)	0.007
Age	<35 years*						
	≥35 years	2.780	16.119 (3.513–73.955)	<0.001	2.485	11.996 (2.094–68.742)	0.005
BMI at delivery	<24 kg/m^2^*						
	≥24 kg/m^2^	1.513	4.542 (2.842–7.261)	<0.001	1.276	3.582 (1.948–6.586)	<0.001
Duration of first stage of labor	<480 min*						
	≥480 min	0.877	2.404 (1.538–3.759)	<0.001	0.225	1.253 (0.682–2.299)	0.467
Duration of second stage of labor	<120 min*						
	≥120 min	2.024	7.571 (3.678–15.582)	<0.001	1.209	3.350 (1.232–9.107)	0.018
Episiotomy	No*						
	Yes	1.563	4.774 (2.989–7.626)	<0.001	0.069	1.071 (0.351–3.272)	0.904
Perineal laceration	No*						
	Yes	1.183	0.306 (0.193–0.487)	<0.001	0.140	1.150 (0.432–3.064)	0.780
Calcium	≥2.1 mmol/L*						
	<2.1 mmol/L	0.229	1.258 (0.499–3.171)	0.627	0.687	1.989 (0.616–6.417)	0.250
Forceps delivery	No*						
	Yes	3.180	24.040 (13.075–44.202)	<0.001	2.956	19.218 (8.347–44.248)	<0.001

* indicates the control group.

Multivariate logistic regression analysis, after adjusting for confounding factors such as age, BMI at delivery, the use of forceps for assisted delivery, and the duration of labor stages, indicated that low albumin levels remained significantly associated with the risk of LAMA, with an odds ratio (OR) of 2.286 (95% CI: 1.259–4.152). Furthermore, the use of forceps for assisted delivery, BMI ≥24 kg/m² before delivery, age ≥35 years, and second stage of labor duration ≥120 min were identified as independent risk factors for LAMA in parturients, with statistically significant differences (all *P* < 0.05). The first stage of labor, episiotomy, perineal tear, and calcium ion levels were not statistically significant (*P* > 0.05), as presented in Table 2.

## 4 Discussion

This study analyzed the clinical data of 410 parturients to explore the impact of low albumin levels on the risk of LAMA and found that low albumin levels significantly increased the risk of LAMA. These results provide crucial evidence for clinical prevention and intervention measures. Receiver operating characteristic analysis was performed to evaluate the predictive accuracy of albumin levels for levator ani muscle rupture. The area under the curve of 0.68 (95% CI: 0.61–0.75) indicates that albumin levels have a moderate predictive value for LAMA, providing additional support for its clinical utility. In actual clinical practice, pregnant women may face various other comorbid infections, such as COVID-19 and Zika virus infections. These infectious conditions can have significant effects on the overall health of pregnant women, indirectly affecting the integrity and function of the levator ani muscle. For instance, Gullo et al. [9] pointed out that Zika virus infection is not only closely associated with congenital abnormalities like microcephaly in fetuses but may also have widespread impacts on the physiological functions of pregnant women. Similarly, Maranto et al. emphasized that COVID-19 infection can lead to more severe disease manifestations in pregnant women, including higher rates of preterm birth and cesarean delivery [10]. Despite the fact that this study did not directly investigate the specific impact of these comorbid infections on prenatal albumin levels and postpartum levator ani muscle rupture risk, it is reasonable to speculate that such infections may indirectly increase the risk of postpartum levator ani muscle rupture by affecting maternal nutritional status, immune function, and inflammatory response. Through our analysis, we discovered a significant correlation between low albumin levels and LAMA, which not only expands the range of risk factors for postpartum LAMA but also offers new targets for early screening and intervention in clinical practice.

The main innovation of this study lies in systematically revealing the potential mechanism underlying the association between albumin levels and LAMA from a biochemical perspective, providing a new dimension to traditional risk factor analysis. Previous studies have primarily focused on obstetric-related factors (such as mode of delivery, fetal weight, parity, etc.) [11–13], while biochemical factors, such as nutritional status, inflammatory status, and tissue repair capacity, have rarely been included in the analysis. This aligns with the role of albumin as a marker of tissue repair and anti-inflammation in existing research [14]. Compared with previous studies, our results fill the gap between maternal systemic status (such as nutritional level) and local injury, laying the foundation for a deeper understanding of the mechanisms underlying LAMA. Furthermore, the results of this study have clear clinical translational implications. Albumin levels, as a serum biochemical indicator, are convenient and cost-effective to measure and can be easily included in routine women’s health check-ups. By identifying high-risk individuals with low albumin levels in a timely manner, clinicians can detect potential issues. They can then implement targeted nutritional supplementation and rehabilitation intervention measures during preconception and pregnancy. This, in turn, will help reduce the incidence of LAMA and related pelvic floor dysfunction.

While this study did not directly assess nutritional interventions’ impact on LAMA risk, albumin levels highlight nutrition’s critical role in postpartum muscle integrity. Emerging research underscores nutrition’s preventive potential: a randomized trial found Boswellia, betaine, and inositol reduced breast fibroadenoma volume by 17.86% (vs 5.96% in controls), modulating inflammatory pathways [14]. Another study suggested inositol and melatonin may enhance ovarian function, warranting exploration of multi-target nutritional strategies for breast cancer prevention [16].

Nutritional supplementation, especially the intake of vitamin D, calcium, and vitamin C, is vital for pelvic floor health. The combination of vitamin D and calcium enhances musculoskeletal function, and their deficiencies are associated with increased risk of pelvic floor dysfunction [17]. Vitamin C, as a key cofactor in collagen synthesis, promotes pelvic floor muscle repair and wound healing, maintaining muscle structure and functional integrity. The synergistic effects of these three nutrients help prevent pelvic floor dysfunction and accelerate postoperative recovery.

Robotic-assisted pelvic floor reconstruction (RARS) repairs core pelvic support structures such as the pubocervical fascial anchor, demonstrating significant advantages over traditional methods in trauma control (40% shorter incisions), functional recovery (35% increase in voluntary voiding rates), and complication prevention (2.3% mesh erosion rate) [18]. For mild to moderate prolapse, conservative treatment (pelvic floor muscle training combined with pessary) is the first choice, with behavioral interventions (e.g., 10% weight loss) reducing prolapse risk by 32% [18]. Surgical intervention is required for moderate to severe cases, and postoperative multidisciplinary rehabilitation after RARS reduces the 3-year reintervention rate to 4.2% [19]. In managing urinary incontinence, anticholinergic drugs are the first-line choice, with high-frequency sacral nerve stimulation (10 kHz) showing 68% efficacy when medications fail, and tibial nerve stimulation reducing urinary leakage by ≥50% in 53% of patients [20]. Laganà et al. confirmed that urinary incontinence patients experience a 37% decrease in sexual function scores and a 41% decline in quality of life, highlighting the need for multidisciplinary management [21]. Song et al. found that childbirth injuries reduce urethral nerve innervation by 42%, providing pathological evidence for preventive strategies and suggesting the need to optimize obstetric management of delivery-related trauma [22].

Zaami et al.’s retrospective analysis showed that only 41% of breast cancer patients received fertility counseling, with 43% choosing preservation measures, indicating a more urgent need among young, nulliparous patients [23]. These data suggest that clinical practice should strengthen standardized fertility preservation procedures. For gestational medication use, Perelli et al. recommend strictly following risk stratification principles, prioritizing low-osmolar contrast agents when necessary, and controlling exposure time windows [24]. Therefore, in exploring future fertility treatments, it is imperative to balance individual differences, overall health status, and the potential of innovative approaches like waterbirth, while maintaining a vigilant stance toward associated risks and uncertainties.

The results of this study complement previous related research to some extent and also reveal new scientific questions. Some studies have confirmed that vaginal delivery and fetal weight greater than 4 kg are important risk factors for LAMA [15]. However, these studies did not fully consider the maternal systemic nutritional status and chronic inflammatory conditions. Building on this, this study further uncovers the link between albumin levels, a systemic indicator, and local tissue injury, providing new insights into understanding the pathogenesis of LAMA. Consistent with previous research findings, this study supports the crucial role of soft tissue resilience and repair capacity in LAMA. For example, studies have shown that the quality and tensile strength of tissue collagen fibers are closely related to the integrity of pelvic floor muscles [25]. This study found that albumin levels may influence collagen fiber remodeling, inflammatory response regulation, and cell metabolism during tissue repair processes, providing a biochemical basis for the occurrence of LAMA. Additionally, previous studies have demonstrated that low albumin levels are associated with an increased risk of postoperative complications and delayed wound healing, which is highly consistent with our finding of a high risk of LAMA in individuals with low albumin levels [26,27]. However, unlike previous studies, our research also presents a new perspective with significant clinical implications: even when serum albumin levels have not yet reached the clinical diagnosis of “hypoalbuminemia,” lower values may still have adverse effects on tissue function. This suggests that traditional diagnostic criteria may need to be further optimized to meet the health needs of specific populations. This study provides valuable evidence for the development of future screening and early intervention strategies.

The research results indicate that parturients with lower albumin levels are more prone to levator ani muscle rupture, which may be closely related to the various biological functions of albumin. First, albumin is an important antioxidant and anti-inflammatory factor in the body, capable of protecting cells and tissues by scavenging free radicals and inhibiting inflammatory responses under stress conditions [28]. This function is particularly crucial during the postpartum tissue repair process. Levator ani muscle rupture is often accompanied by local tissue inflammation and repair processes, and lower albumin levels may lead to excessive local inflammatory responses and delayed repair, thereby increasing the risk of tissue rupture [7]. Second, albumin level is a reliable indicator of the body’s nutritional status. Low albumin levels usually suggest malnutrition or chronic wasting conditions, which may result in decreased soft tissue resilience and regenerative capacity [29]. In postpartum women, nutritional demands increase, and parturients undergoing cesarean section or experiencing significant hemorrhage are more likely to have decreased albumin levels. Studies have shown that disorders in collagen metabolism are closely associated with reduced pelvic tissue elasticity, and as a key factor in maintaining collagen synthesis, decreased albumin levels may further exacerbate the fragility of pelvic muscles [30,31]. Furthermore, lower albumin levels may affect processes such as cell proliferation and angiogenesis, leading to decreased local tissue healing capacity [32]. It is important to note that this study found that even when albumin levels are within the low end of the normal range, they still have a significant impact on the risk of levator ani muscle rupture. This phenomenon suggests that the current normal reference value range may need to be adjusted based on the actual physiological needs of specific populations. Meanwhile, this result emphasizes that in clinical practice, reliance solely on diagnostic thresholds for single indicators is inadequate; instead, individualized assessments and dynamic monitoring of changes in albumin levels should be incorporated. Other related studies have pointed out [33,34] that fetal abdominal circumference and fetal weight are risk factors for postpartum levator ani muscle injury, and vaginal trial of labor in parturients undergoing cesarean section may increase the area of levator ani muscle rupture after pelvic organ prolapse.

In vitro fertilization (IVF) conception may exacerbate the risk of postpartum levator ani avulsion in women with hypoalbuminemia by disrupting hormonal balance and endometrial environment. Insufficient albumin impairs muscle repair capacity, and this risk becomes particularly pronounced during IVF pregnancies. Studies indicate that IVF women face elevated risks of gestational complications (including pelvic floor dysfunction) due to factors such as advanced maternal age and hormonal therapies, with hypoalbuminemia further aggravating muscle damage risks [35]. Additionally, cesarean section history and scar defects may also affect IVF pregnancy outcomes, demonstrating the complex influence of prior pregnancy experiences on gestational outcomes in IVF patients. Future research should investigate risk mechanisms and intervention measures for IVF-conceived women (especially those with hypoalbuminemia), focusing on the interaction between IVF and hypoalbuminemia, as well as the impact of prior pregnancies on pelvic floor function.

However, this study also has certain limitations. First, the sample size is relatively small and the study is single-center, which may limit the statistical power of the results and their generalizability. Multi-center, large-sample studies would better validate the conclusions of this study. Second, this study is retrospective in design, with potential biases and confounding factors that may not have been fully controlled. Future research should be based on larger prospective studies to further validate the conclusions of this study and explore the potential biological mechanisms between low albumin levels and levator ani muscle rupture. Additionally, although the study has attempted to control for confounding factors, there may still be unaccounted-for interfering variables, such as other systemic diseases or socioeconomic factors. Finally, the study did not further explore the molecular mechanisms of albumin’s action, such as its interactions with collagen metabolism and cellular inflammatory factors, which remains an area for future in-depth research.

Furthermore, considering the findings of this study, the selection of future fertility treatment options for patients with low albumin levels requires careful consideration. A comprehensive preoperative assessment of the patient’s overall health status is crucial, particularly for procedures that may require more personalized considerations, such as colposacropexy. Gullo et al. highlighted the importance of precision medicine based on molecular characteristics in fertility treatment [36]. For patients with low albumin levels, analyzing their molecular features may contribute to the development of more individualized treatment plans, thereby enhancing treatment efficacy and reducing complications. Additionally, Vidiri et al. emphasized that when evaluating any fertility treatment options, it is essential to thoroughly consider the risks associated with the procedure and the postoperative recovery [37]. For patients with low albumin levels, postoperative rehabilitation and nutritional support are particularly important to facilitate their rapid recovery. Notably, for patients who have undergone complex deliveries, auxiliary methods such as waterbirth may offer potential benefits in alleviating pain and promoting recovery, though its long-term effects and risks remain to be further elucidated through high-quality research.

In conclusion, this study confirms low albumin levels as an independent risk factor for levator ani muscle rupture, closely associated with maternal age, BMI, and delivery process. These findings provide an effective risk assessment tool for clinical practice, assisting healthcare professionals in making informed decisions during prenatal management. Future research should focus on how to utilize these results to improve maternal management strategies, reduce the incidence of levator ani muscle rupture, and further enhance maternal health and delivery safety.
